# Probiotic supplementation during pregnancy alters gut microbial networks of pregnant women and infants

**DOI:** 10.3389/fmicb.2022.1042846

**Published:** 2022-12-01

**Authors:** Ting Huang, Zhe Li, Kian Deng Tye, Sze Ngai Chan, Xiaomei Tang, Huijuan Luo, Dongju Wang, Juan Zhou, Xia Duan, Xiaomin Xiao

**Affiliations:** ^1^Department of Obstetrics and Gynecology, The First Affiliated Hospital of Jinan University, Guangzhou, China; ^2^Department of Obstetrics and Gynecology, The Third Affiliated Hospital of Sun Yat-sen University, Guangzhou, China; ^3^Department of Obstetrics and Gynecology, The Fifth Affiliated Hospital of Guangzhou Medical University, Guangzhou, China

**Keywords:** probiotics, pregnancy, gut microbiota, microbial network, 16S rRNA, community stability

## Abstract

**Background:**

Probiotic supplementation has been popular and widespread, yet we still lack a comprehensive understanding of how probiotic supplementation during pregnancy affects the gut microbial networks of pregnant women and infants. In this study, we firstly used network analysis to compare the gut microbiota of pregnant women with and without probiotic supplementation, as well as their infants.

**Methods:**

Thirty-one pairs of healthy pregnant women and infants were recruited and randomly divided into the probiotic group (15 mother-infant pairs) and the control group (16 mother-infant pairs). Pregnant women in the probiotic group consumed combined probiotics from 32 weeks to delivery. Fecal samples were collected from pregnant women and infants at several time points. Gut microbiota was evaluated using 16S rRNA gene sequencing. Intestinal microbial network and topological properties were performed using the molecular ecological network analysis.

**Results:**

No significant difference was found between the probiotic and control groups on the microbial alpha and beta diversity. As the gestational age increased, the total links, average degree, average clustering coefficient, robustness, and the proportion of positive correlations were increased in pregnant women with probiotics administration. In contrast, these indices were decreased in infants in the probiotic group.

**Conclusion:**

Probiotic supplement does not change the microbial diversity of pregnant women and infants, but significantly alters the intestinal microbial network structure and properties. Although pregnant women have more complicated and stable networks after probiotic administration, their infants have less stable networks.

## Introduction

Gut microbiota plays a key role in host immune, physiological, and pathological processes. Dysbiosis of the gut microbiota affects disease initiation and progression, such as pregnancy complications, adverse pregnancy outcomes, metabolic diseases, immune-related diseases, neurologic and psychiatric diseases (Wang et al., 2019; Gomaa, 2020; Hasain et al., 2020). Therefore, it is significant to explore the microorganisms that inhabit human intestines.

Probiotics are considered as living microorganisms that can be beneficial to the host (Sanders, 2008; Hill et al., 2014), which could modulate imbalanced microbial communities and human health. The consumption of probiotics become popular in recent years. Furthermore, probiotic, as a non-prescription drug in China, is available in pharmacies and online stores. Some pregnant women may purchase their own probiotic tablets or yogurt. In our previous study, we found that probiotic supplementation did not influence the composition of the gut microbiota and the corresponding bacteria of ingested probiotics (Chen et al., 2019). However, other gut bacteria changed in pregnant women who took probiotics (Chen et al., 2019). We speculated that probiotics may act not by increasing their abundance, but rather by influencing the overall gut microbial community and interactions. The current research on probiotics has focused primarily on the changes in the abundance of intestinal bacteria. The potential role of probiotics on microbial interactions remains unclear, and requires further research. Additionally, research showed that early life microbial colonization may begin *in utero* (Al Alam et al., 2020), and whether probiotic administration during pregnancy alters infant gut microbiota remains unknown. Consequently, it is necessary to examine the influence of probiotic supplementation during pregnancy on infant gut microbiota.

Most intestinal microbial studies paid attention to dominant microbes, microbial diversity, and the identification of biomarkers. However, microbes do not exist in isolation, their interactions play an important role in maintenance of microbial community homeostasis. Fewer studies explored intestinal microbial interactions. Network analysis could help us understand microbial interactions and community structure comprehensively (Fuhrman, 2009; Faust and Raes, 2012). Therefore, network analysis is suitable for us to evaluate the effect of probiotics supplementation on intestinal microbiota interactions. Molecular ecological network analysis (MENA) is an algorithm that is capable of automatically determining an appropriate threshold for constructing a network (Deng et al., 2012). And MENA’s results are more objective than those of other network analysis algorithms that determine the threshold artificially. Recently, MENA has been used to assess complex microbial communities in humans (Cong et al., 2019), animals (Xiao et al., 2022), lakes (Liu et al., 2022), and soils (Yuan, 2021).

In this study, we constructed MENA networks for pregnant women with and without probiotic supplement and their infants. The primary aim was to determine whether probiotic administration during pregnancy affects infant gut microbiota structure and development. Additionally, we also analyzed the alteration of microbial networks in pregnant women. We assessed alterations from the aspect of time and probiotic intervention. This study offers new insight into the intestinal microbiota of pregnant women and infants response to probiotics and provides a reference for the application of probiotics.

## Materials and methods

### Participants and study design

**FIGURE 1:**
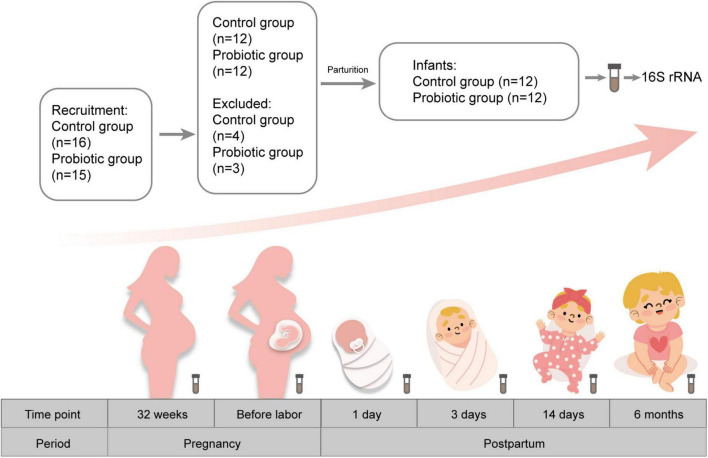
Flow chart for screening participants.

This study was conducted at The First Affiliated Hospital of Jinan University (Guangzhou, China). Pregnant women were recruited before 32 weeks of gestation. Exclusion criteria were pregnancy complications, multiple pregnancy, vaginitis, preterm birth, and other chronic diseases. A total of 31 pregnant women were recruited in this study and were randomly assigned to the probiotic group (*n* = 15) and the control group (*n* = 16). During follow-up, three women in the probiotic group were excluded due to obesity, irregular medication, and lost to follow-up. In the control group, four women were eliminated (including one with gestational hypertension, one with threatened premature birth and two lost to follow-up). Finally, 24 pregnant women were enrolled in this study (Figure 1). Women in the probiotic group took two probiotic tablets (Golden Bifid, from Inner Mongolia Shuang qi Pharmaceutical Company, China) twice a day until delivery. They were instructed to return the packages of tablets to evaluate their compliance, and the total number of unused tablets was < 10%. Golden Bifid consisted of tablets containing 5 million live bacteria of *Bifidobacterium longum* (0.5 × 10^7^ CFU), *Lactobacillus delbrueckii bulgaricus* (0.5 × 10^6^ CFU), and *Streptococcus thermophilus* (0.5 × 10^6^ CFU). Participants who take antibiotics or other probiotic products during the experiment were excluded. Meanwhile, infants were also enrolled in this study. All infants in our study were exclusively breastfed until 4 months of age. Then they begin adding complementary foods to the diet. Participants were asked for feedback on the episodes of gastrointestinal symptoms during the follow-up period. The baseline characteristics, such as maternal age, BMI, and gestational weeks at delivery, were obtained from electronic medical records.

This study was approved by the Institutional Review Board for Human Subject Research at The First Affiliated Hospital of Jinan University and conducted according to the regulations of the Declaration of Helsinki. All women have signed informed consent before participating in this study.

### Sample collection

We collected fecal samples from each pregnant woman before probiotic supplementation (32 weeks of gestation) and labor began, respectively. Additionally, fecal samples series including infants aged 1 day, 3 days, 14 days, and 6 months were also collected. Fecal samples were divided into 12 groups: feces from pregnant women in the control group at the first collection and the second collection (PC1, PC2), feces from pregnant women in the probiotic group at the first collection and the second collection (PP1, PP2), feces from infants in the control group at day 1, 3, 14 and month 6 after birth (CD1, CD3, CD14, CM6), feces from infants in the probiotic group at day 1, 3, 14 and month 6 (PD1, PD3, PD14, PM6) after birth. One sample in the PP2 group and another in the PD14 group were missed because the participants were absent at the time of planned collection and women failed to collect the sample despite being reminded. A total of 142 samples were finally collected. Fecal samples were collected in sterile tubes and stored at –80°C until sent for sequencing.

### DNA extraction, sequencing, and species annotation

Total bacterial DNA was extracted from sample using CTAB/SDS technique. DNA concentration and purity were monitored on a 1% agarose gel. We used a specific primer (515F/806R) to amplify the V4 hypervariable region of the 16S rRNA gene in each sample. All PCR reactions were carried out with Phusion^®^ High-Fidelity PCR Master Mix (New England Biolabs). The PCR products were mixed with the same volume of 1X loading buffer (containing SYB Green) and electrophoresed on a 2 percent agarose gel. PCR products were mixed in equal density ratio. The mixed PCR products were then purified using the GeneJET Gel Extraction Kit (K0692, Thermo Scientific, USA). Sequencing libraries were generated by using the Ion Plus Fragment Library Kit 48 rxns (Thermo Scientific). The library was sequenced on a Ion S5™ XL platform after assessing on the Qubit@ 2.0 Fluorometer. Clean reads were obtained after data split, filtration, and chimera removal. Uparse software were used to cluster sequences into operational taxonomic units (OTUs) at a 97% similarity threshold. Finally, representative sequences for each OTU were annotated taxonomic information based on the Silva Database.

### Microbial diversity analysis

Shannon index and Simpson index were calculated to evaluate alpha diversity. Differences in Shannon and Simpson indices between groups were tested with one-way ANOVA. Principal coordinates analysis (PCoA) was performed based on the unweighted UniFrac distances to assess community diversity. Alpha diversity and unweighted UniFrac distances were calculated with QIIME software (Version 1.9.1) and displayed with RStudio software (Version 1.4.1717).

### Co-occurrence network construction and topological properties

To avoid interference with the results by rare species or species that contaminate the samples, OTUs presented in less than 50% of samples were filtered before constructing the microbial network. Co-occurrence networks were generated using the Molecular Ecological Network Analysis Pipeline (MENAP)^1^ based on log-transformed OTU relative abundance (Zhou et al., 2010; Deng et al., 2012). The adjacency matrix was generated by a random matrix theory (RMT)-based approach. The advantage of MENAP is that the threshold for network construction is automatically determined on the basis of an RMT-based approach (Deng et al., 2012). This method could avoid the inaccuracy of network results generated by arbitrary thresholds. The microbial networks were visualized by Gephi (Version 0.9.2) or Cytoscape (Version 3.7.1).

Network topological properties, including the number of nodes, links, average degree, average clustering coefficient, average path distance, and modularity, were calculated in the MENAP. To prove the significance of the molecular ecological networks, we constructed 100 random networks. The mean and standard deviation of network topological properties from the 100 random networks were calculated and then compared with molecular ecological networks.

### Network stability analysis

To assess the gut microbial network stability, robustness was carried out as described by Yuan et al. (Yuan, 2021). A network’s robustness is measured by the percentage of species left in the network following the removal of random or targeted nodes (Dunne et al., 2002; Montesinos-Navarro et al., 2017). Microbial communities with higher robustness are more stable. In this study, robustness was calculated after randomly removing 50% of nodes or 50% of potential keystones. Potential keystones were identified according to Guimerà (Guimerà and Nunes Amaral, 2005).

### Statistical analysis

The significance of baseline characteristics between the control group and the probiotic group was assessed using the Student’s *t*-test or Chi-square test. The comparison of robustness between groups was evaluated by the one-way ANOVA. Statistical differences between groups were analyzed using Graphpad Prism software (Version 8.0.2).

## Results

### Characteristics of participants

The characteristic of the participants were presented in Table 1. All clinical data showed no significant difference between the two groups. The duration of probiotic administration was 52 ± 7.08 days. The mothers and their infants did not report any gastrointestinal symptoms until the end of the follow-up.

**TABLE 1:** Characteristics of participants.

	Control group (*n* = 12)	Probiotic group (*n* = 12)	*P*-value

Maternal characteristics			
Age (year)	27.33 ± 2.90	27.42 ± 3.09	0.95
Prepregnancy BMI (kg/m^2^)	20.38 ± 2.69	21.00 ± 2.90	0.59
BMI at delivery (kg/m^2^)	25.00 ± 2.52	25.88 ± 2.86	0.43
Gestational days (days)	278.33 ± 4.68	276.00 ± 7.40	0.37
Delivery mode			0.58
Vaginal, *n*	11	9	
Cesarean, *n*	1	3	
No smoking during pregnancy, *n*	12	12	
No drinking during pregnancy, *n*	12	12	
Education level			0.38
High school, *n*	2	1	
University, *n*	10	11	

**Infant characteristics**			

One-minute Apgar score	9	9	
Gender			0.67
Male, *n*	5	3	
Female, *n*	7	9	
Weight at birth (kg)	3.27 ± 0.44	3.38 ± 0.48	0.54
Length at birth (cm)	49.92 ± 1.68	49.83 ± 1.75	0.91
BMI at birth (kg/m^2^)	13.05 ± 1.05	13.56 ± 1.09	0.26
Head circumference (cm)	36.25 ± 8.18	33.83 ± 1.03	0.33

### Microbial diversities were not changed after probiotic supplementation

Shannon index, Simpson index, and PCoA were performed to assess microbial diversity between the probiotic group and the control group. There were no significant differences on Shannon and Simpson index of pregnant women between the two groups (Supplementary Figures 1A,B). PCoA demonstrated that fecal samples from the same period clustered together (Supplementary Figure 1C). Infant alpha diversity fluctuated over time, but no significant change between the control group and probiotic group at the same time point (Supplementary Figures 1D,E). As shown in Supplementary Figure 1F, microbial communities were divided into three clusters according to time periods. These results indicated that the intestinal microbial diversity of pregnant women was not influenced by probiotic intervention. Infant microbial diversity altered with increasing infant age instead of maternal probiotic supplementation.

### Microbial networks and topological properties of pregnant women and infants

**FIGURE 2:**
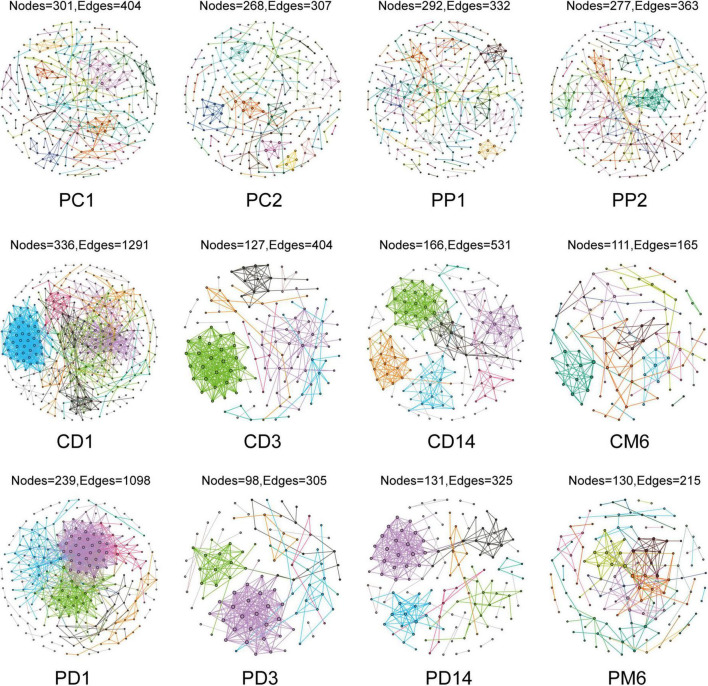
Overview of the networks in pregnant women and infants. Each node represents one OTU, edges indicate significant correlations between OTUs. Different color represents different modules. PC1 and PP1 represent feces from pregnant women in the control group and the probiotic group at the first sampling time, respectively; PC2 and PP2 represent feces from pregnant women in the control group and the probiotic group at the second sampling time CD1, CD3, CD14, and CM6 represent feces from infants in the control group at day 1, 3, 14, and month 6 after birth, respectively; PD1, PD3, PD14, and PM6 represent feces from infants in the probiotic group at day 1, 3, 14, and month 6 after birth, respectively.

Network analysis could illustrate interactions between microbes and provide novel insight to explore community structure. We constructed co-occurrence networks to compare gut microbiota in the control group and the probiotic group by using MENA methods (Figure 2). All networks demonstrated scale-free features under the same similarity threshold (0.860), with *R*^2^ of power-law ranging from 0.725 to 0.929 (Supplementary Table 1), indicating that most nodes have few links, whereas few nodes have many links (Barabási, 2009). Meanwhile, empirical networks had higher clustering coefficients and modularity indices than their corresponding random networks (Supplementary Table 1), which implied that empirical networks are non-random.

**FIGURE 3:**
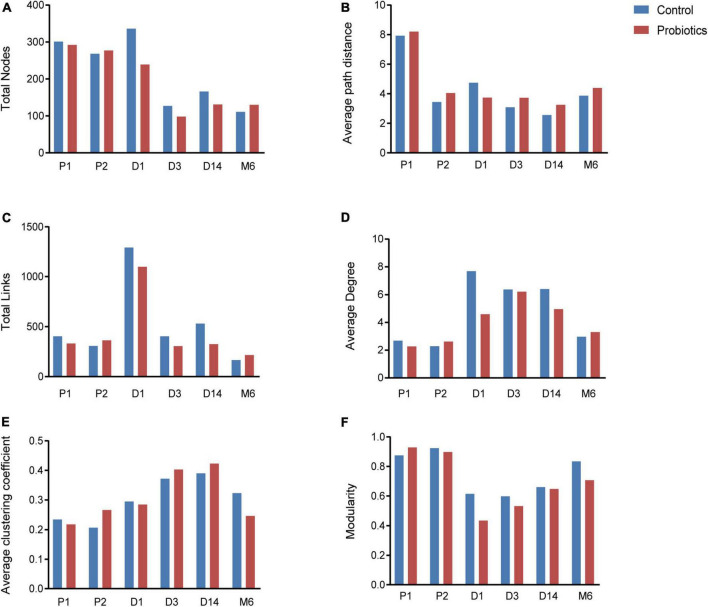
Topological properties of microbial networks, including total nodes **(A)**, average path distance **(B)**, total links **(C)**, average degree **(D)**, average clustering coefficient **(E)**, and modularity **(F)**. P1 = Fecal samples of pregnant women at the first time collection; P2 = Fecal samples of pregnant women at the second time collection; D1 = Infant fecal samples collected at day 1 after birth; D3 = Infant fecal samples collected at day 3 after birth; D14 = Infant fecal samples collected at day 14 after birth; M6 = Infant fecal samples collected at month 6 after birth.

Microbial networks and several topological properties of pregnant women with and without probiotic supplementation had different trends over time. The total nodes and average path distance of pregnant women in both the control group and probiotic group decreased with the increasing gestational age (Figures 3A,B). The number of links, average degree, and average clustering coefficient of pregnant women in the control group decreased with gestational weeks (Figures 3C–E), suggesting that intestinal microbial networks of healthy pregnant women become less complex as gestational age increases. In contrast, these topological properties increased among pregnant women receiving probiotics (Figures 3C–E). These results suggested that microbial networks of pregnant women become more complicated and tighter in response to probiotics.

Characteristics of microbial community networks varied across infant developmental stages. Specifically, 1-day-old infant networks contained more nodes and links (Figures 3A,C), which increased the complexity of networks. The 14-day-old infants had cohesive microbial networks, which was reflected by the shorter average path distance and higher clustering coefficient (Figures 3B,E). As infants grew, their network modularity indices increased and were close to that of their mothers (Figure 3F). Notably, although infants in the probiotic group showed similar changes in topological features as the control group, there were differences in the values of topological features at the same period between the two groups. For instance, infants in the probiotic group had lower complicated networks than those in the control group at the first three sampling time points (Figures 3C,D). In addition, modularity indices of infants in the probiotic group were lower than those in the control group at each sampling time point (Figure 3F). These findings revealed that maternal probiotic supplementation may not alter overall trends in infant network topological properties over time, but decrease the network complexity and modularity.

### Modularity and interactions of microbial networks analysis

**FIGURE 4:**
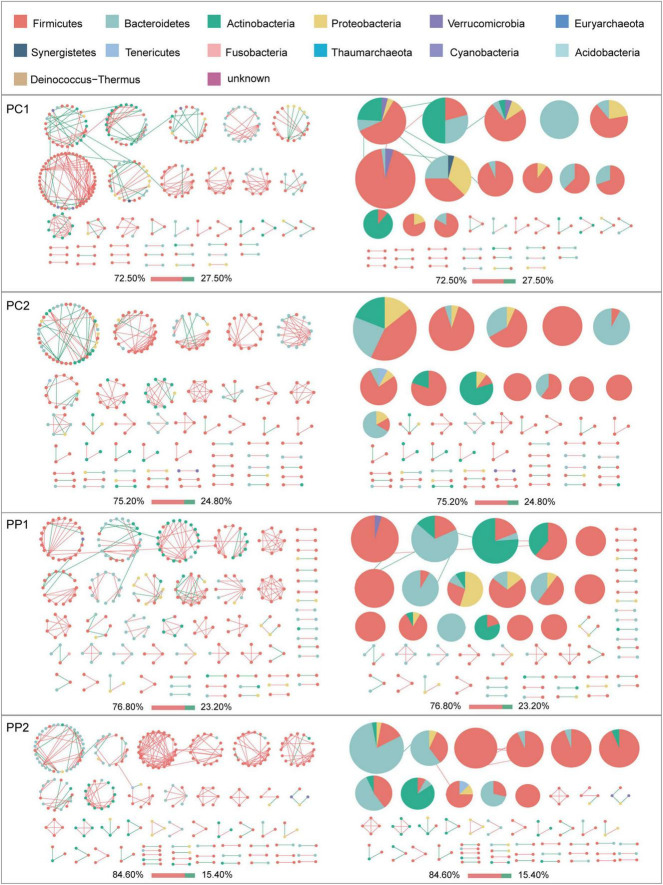
Modules within gut microbial networks of pregnant women with or without probiotic supplementation. The colors of nodes represent different major phyla; pie charts exhibit the phylum level composition of modules with ≥ 5 nodes. Red edges indicate positive correlations between nodes, whereas green edges indicate negative correlations. The bar underneath each networks demonstrate the proportion of positive and negative correlations.

**FIGURE 5:**
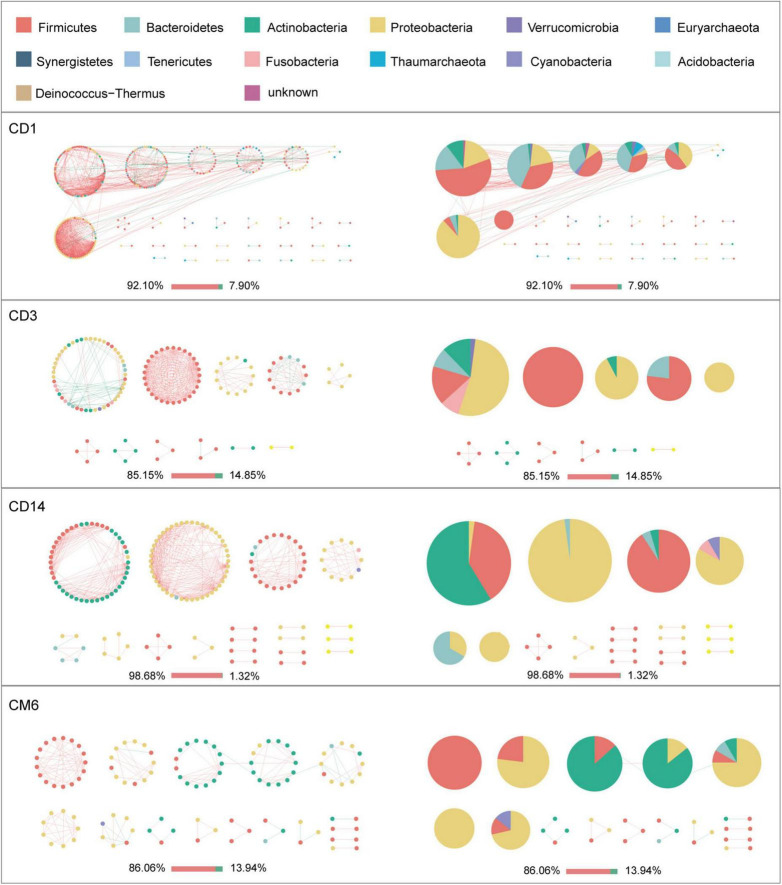
Modules within gut microbial networks of infants in the control group. The colors of nodes represent different major phyla; pie charts exhibit the phylum level composition of modules with ≥ 5 nodes. Red edges indicate positive correlations between nodes, whereas green edges indicate negative correlations. The bar underneath each networks demonstrate the proportion of positive and negative correlations.

**FIGURE 6:**
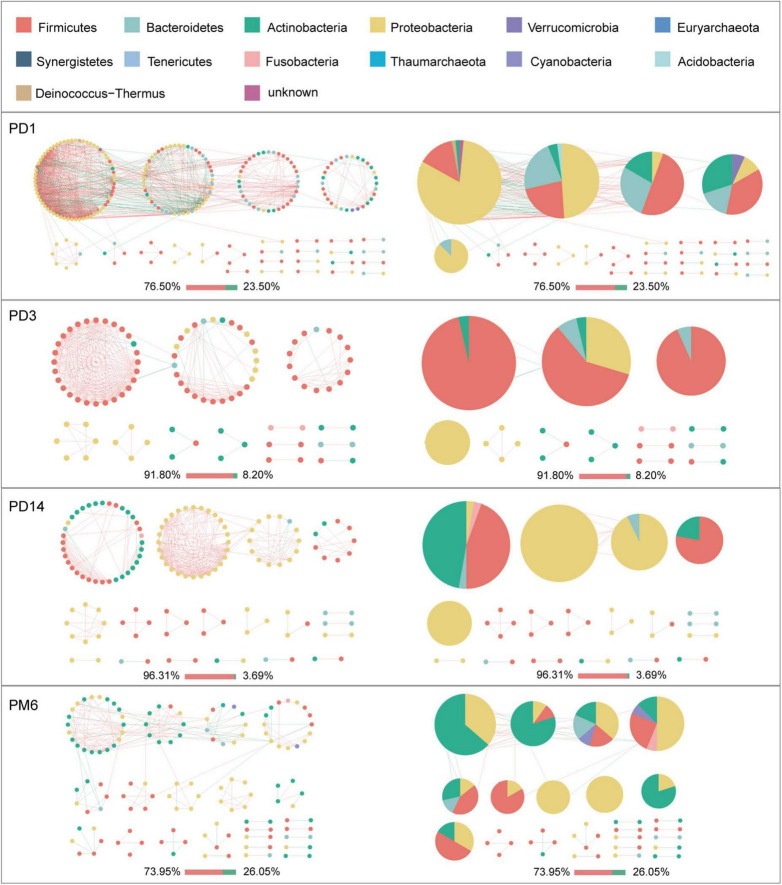
Modules within gut microbial networks of infants in the probiotic group. The colors of nodes represent different major phyla; pie charts exhibit the phylum level composition of modules with ≥ 5 nodes. Red edges indicate positive correlations between nodes, whereas green edges indicate negative correlations. The bar underneath each networks demonstrate the proportion of positive and negative correlations.

Given the modularity of networks was changed in participants in the probiotic group, we divided all network nodes into different modules according to modularity and highlighted large modules (at least five nodes) (Figures 4–6). Then, we analyzed interactions between microbes and taxonomic composition of modules. Overall, microbes tended to co-occur in all networks (72.50–98.68%), but probiotic supplement changed the proportion of positive correlations in both pregnant women and infants. More specifically, the proportion of positive correlations increased by 7.8% in the probiotic group with increasing gestational age, while it only increased by 2.7% in the control group (Figure 4). In infant microbial networks, a lower proportion of positive correlations was observed in the probiotic group at all periods compared to the control group (76.5 vs. 92.10%, 96.31 vs. 98.68%, and 73.95 vs. 86.06%), except for day 3 after birth (Figures 5, 6). Collectively, the above results indicated that probiotic administration during the third trimester may enhance women’s intestinal microbial symbiosis, but weaken infants’ microbial symbiosis.

In terms of the taxa composition of modules, *Firmicutes* presented in almost all large modules of women’s networks, and it was a prominent phylum in most large modules. Of note, the number of large modules in the probiotic group decreased by seven as gestational weeks increased, whereas there was no obvious change in the control group, suggesting that probiotic supplementation may reduce network modularity. This result corresponds to the above modularity index (Figure 3F). In aspect of infant network modules, a similar trend was observed in number of modules between the probiotic and control groups over time. The number of modules in both groups of infants was lowest on day three and then increased with age (Figures 5, 6). However, the dominant phyla of infant modules changed with time and maternal probiotic intervention. At the first sampling, *Firmicutes* dominated most modules in the control group, while *Proteobacteria* was a prominent member of the large modules in the probiotic group. At the second sampling, the proportion of *Proteobacteria* in module composition of the control group increased, whereas a higher proportion of *Firmicutes* was observed in the probiotic group. At the last two samplings, *Firmicutes*, *Proteobacteria*, and *Actinobacteria* dominated various modules in both the control group and the probiotic group.

### Stability of microbial community networks

**FIGURE 7:**
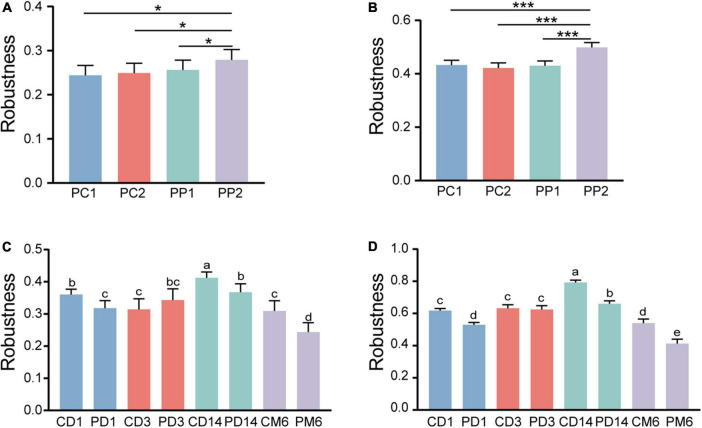
Microbial network stability of pregnant women and infants. Robustness of pregnant women is measured as the proportion of the remaining taxa after **(A)** randomly removing 50% of nodes and **(B)** 50% of potential keystones taxa. Statistical significance was determined by the one-way ANOVA. *P < 0.05; ***P < 0.001. Panels **(C,D)** showed the robustness of infants. The calculation method is the same as panels **(A,B)**, respectively. Different lowercase letters above the bars indicate differences with *P* < 0.05, whereas the same letter indicates no significant difference.

To evaluate whether and how microbial network stability of pregnant women alters with probiotic supplementation, robustness was calculated. As pregnancy progressed toward term, there was no significant alteration in robustness of the control group (*P* = 0.99, *P* = 0.49) (Figures 7A,B). However, the robustness of the probiotic group markedly increased, and was higher than that of the control group (*P* = 0.01, *P* < 0.001) (Figures 7A,B). This confirmed that probiotic intake may improve the stability of the gut microbiome network in pregnant women.

We then measured the robustness of infants to investigate whether infants in the probiotic group had more stable microbial networks. Contrary to our expectation, infants in the probiotic group had lower robustness than the control group at three periods (1 day, 14 days and 6 months after birth, *P*-values were all less than 0.01) (Figures 7C,D). The robustness of microbial network at 3 days after birth had no obvious differences among the probiotic group and control group (*P* = 0.18, *P* = 0.97). These results implied that probiotics during pregnancy may reduce the stability of infant gut microbiota. Additionally, we found that a more stable microbial network contained more positive connections (Figures 4–6).

## Discussion

Revealing intestinal microbial interactions and stability are critical but easily ignored issues. Through analyzing molecular ecological networks of gut microbiota from pregnant women and infants, we confirmed that microbial networks of pregnant women and infants will change as time goes on, and probiotic supplementation may interfere with these changes and microbial stability. These results increase our understanding of the influence of probiotics on gut microbiota. Meanwhile, our findings also provide a reference for the clinical application of probiotics.

In our research, no significant differences were found in alpha and beta diversity between the control group and the probiotic group. Several studies also reported that probiotic supplementation does not alter the intestinal microbial diversity of adults (Kristensen et al., 2016; Singh et al., 2018). Additionally, early life probiotic exposure was also confirmed not to change infant gut microbiota diversity (Ismail et al., 2012; Dotterud et al., 2015; Murphy et al., 2019). Our results are consistent with the above findings.

Given no remarkable changes were observed in terms of microbial diversity, we constructed co-occurrence networks of participants in the probiotic group and control group. Although the probiotic is known to exert effects on pregnancy outcomes, its influence on the intestinal microbial networks was first explored in this study. We found that probiotic supplementation may be beneficial to the gut microbiota of pregnant women. For instance, average degree, which represents the complexity of networks, was increased in pregnant women with probiotics supplements. A recent study reported that networks with higher complexity tend to be more stable (Liu et al., 2022). Robustness, an index used to evaluate the stability of networks, was observed elevated in women with probiotics administration. In addition, our results showed that the proportion of positive correlations of pregnant women in the probiotic group was increased. Cooperation may be a major interaction between microbes in a relatively healthy gut (Jain and Krishna, 2001; Ma, 2018; Cong et al., 2019). These findings suggested that women treated with probiotics during the third trimester may have a more stable and healthy microbial network.

Early life periods are critical windows for individual growth and establishment of the immune system. Several studies have demonstrated that gut microbiota colonization may begin *in utero*, and microbial exposure during early life can activate the host immune system (Walker et al., 2017; Mishra et al., 2021). The normal colonization process of microbiota during this period is vital for individual health. Hence, we further explored the effect of maternal probiotic supplementation on infant gut microbiota. Contrary to our expectation, the intestinal microbial networks of infants in the probiotic group were less complex and stable than the control group. In most sampling time points, the probiotic group had a lower proportion of positive correlations than the control group. We speculated that this is because the process of microbiota transmission from mother to infant is complicated and has not been fully explored. Maternal vaginal, skin, oral, gut, uterus, and breast milk communities were reported to contribute to early life microbiota (Collado et al., 2016; Ferretti et al., 2018; Differding and Mueller, 2020). Moreover, probiotics may influence the communities of breast milk and the vagina (Liang et al., 2021; Shin et al., 2021). In this study, it was confirmed that probiotic intake could alter maternal gut microbial network and interactions. Changes in the microbiome at any of the above sites may alter the infant microbiota. Furthermore, we found that infants in the probiotic group had a different dominant phylum of network modules compared to the control group, and their network modularity was lower as well. Recent studies showed that healthy humans have higher modularity of microbial networks than individuals with colorectal cancer or inflammatory bowel disease (Baldassano and Bassett, 2016; Cong et al., 2019). This seems to suggest that a healthy gut microbiome is more modular. Currently, we do not know whether these infants will have different health outcomes in the future, which require long-term follow-up research with a large sample size.

## Limitations

Our study had several limitations. Firstly, this study is limited by its small sample size. Despite this, our results showed significant differences in microbial networks between the control group and the probiotic group. Secondly, we did not record infant weight and height after birth. Long-term follow-up is needed to evaluate the influence of probiotic supplementation during pregnancy on infant growth and long-term health outcomes.

## Conclusion

Probiotic supplementation during the third trimester may not change the microbial diversity of healthy pregnant women and infants but alter their microbial network properties and stability. Although pregnant women have more complicated and stable networks after probiotic administration, their infants have less stable networks. Therefore, we suggest that healthy pregnant women should use probiotics with caution.

## Data availability statement

The datasets presented in this study can be found in online repositories. The names of the repository/repositories and accession number(s) can be found below: https://www.ncbi.nlm.nih.gov/, PRJNA876635.

## Ethics statement

The studies involving human participants were reviewed and approved by the Institutional Review Board for Human Subject Research at The First Affiliated Hospital of Jinan University. Written informed consent to participate in this study was provided by the participants’ legal guardian/next of kin.

## Author contributions

XX conceived and designed the study. TH, ZL, KT, SC, XT, DW, JZ, XD, and HL collected the samples and clinical data. ZL and KT conducted the experiments. TH and SC analyzed the data. DW and JZ described the methodology. TH prepared the manuscript. XT and HL contributed to review of the manuscript. XX supervised the research project. All authors had full access to the final version of the report and agreed to the submission.

## Footnotes

1
http://ieg2.ou.edu/MENA/
