# Supplementary material for: Probiotic supplementation during pregnancy alters gut microbial networks of pregnant women and infants
Source: Front Microbiol. 2022 Dec 1;13:1042846. doi: 10.3389/fmicb.2022.1042846 (PMC9751803; doi:10.3389/fmicb.2022.1042846)
Supplement: Supplementary file 1 [file Data_Sheet_1.docx]

Supplementary Material

# Supplementary Figures


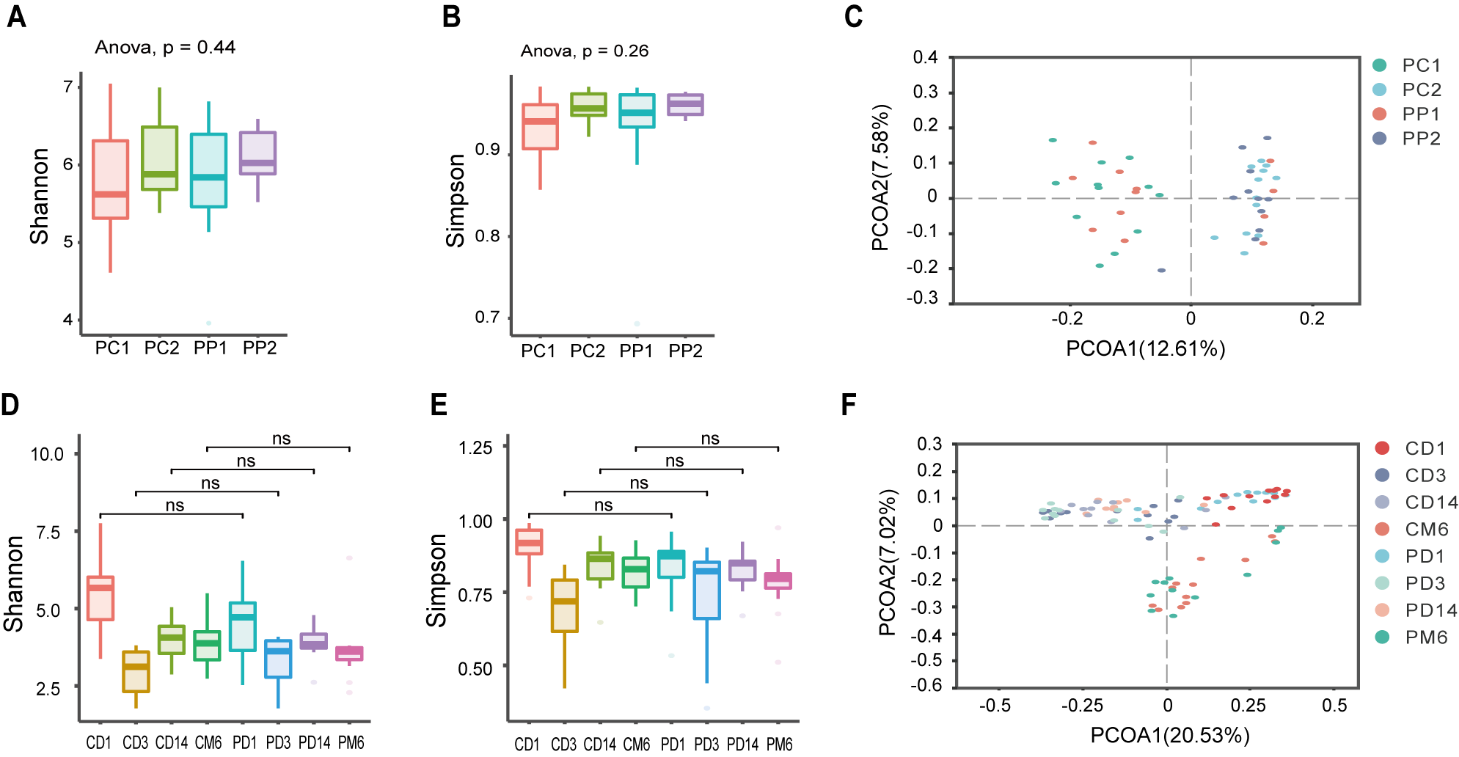


Figure S1: Alpha diversity and Beta diversity of gut microbiota. (A-C) show Shannon index, Simpson index, and PCoA of pregnant women, respectively. (D-F) display Shannon index, Simpson index, and PCoA of infants, respectively.

# Supplementary Tables

Table S1: Topological properties of the networks in pregnant women and infants

|  | PC1 | PC2 | PP1 | PP2 | CD1 | CD3 | CD14 | CM6 | PD1 | PD3 | PD14 | PM6 |
| --- | --- | --- | --- | --- | --- | --- | --- | --- | --- | --- | --- | --- |
| MENA networks | | | | | | | | | | | | |
| Nodes | 301 | 268 | 292 | 277 | 336 | 127 | 166 | 111 | 239 | 98 | 131 | 130 |
| Links | 404 | 307 | 332 | 363 | 1291 | 404 | 531 | 165 | 1098 | 305 | 325 | 215 |
| AD | 2.684 | 2.291 | 2.274 | 2.621 | 7.685 | 6.362 | 6.398 | 2.973 | 9.188 | 6.224 | 4.962 | 3.308 |
| ACC | 0.234 | 0.207 | 0.218 | 0.266 | 0.295 | 0.372 | 0.39 | 0.323 | 0.285 | 0.403 | 0.423 | 0.246 |
| APD | 7.927 | 3.446 | 8.204 | 4.047 | 4.745 | 3.091 | 2.559 | 3.867 | 3.739 | 3.725 | 3.256 | 4.399 |
| Modularity | 0.875 | 0.924 | 0.929 | 0.898 | 0.615 | 0.598 | 0.66 | 0.835 | 0.434 | 0.532 | 0.648 | 0.707 |
| R^2^ of power-law | 0.874 | 0.913 | 0.842 | 0.925 | 0.806 | 0.784 | 0.803 | 0.881 | 0.725 | 0.742 | 0.797 | 0.929 |
| Cutoff | 0.86 | 0.86 | 0.86 | 0.86 | 0.86 | 0.86 | 0.86 | 0.86 | 0.86 | 0.86 | 0.86 | 0.86 |
| Random networks | | | | | | | | | | | | |
| ACC±SD | 0.008±0.004 | 0.006±0.004 | 0.006±0.004 | 0.010±0.004 | 0.081±0.007 | 0.137±0.014 | 0.075±0.008 | 0.029±0.013 | 0.175±0.012 | 0.160±0.019 | 0.090±0.013 | 0.034±0.011 |
| Modularity±SD | 0.665±0.010 | 0.734±0.009 | 0.748±0.008 | 0.669±0.009 | 0.295±0.005 | 0.304±0.007 | 0.331±0.008 | 0.567±0.011 | 0.233±0.005 | 0.291±0.008 | 0.376±0.009 | 0.523±0.011 |

AD: average degree; ACC: average clustering coefficient; APD: average path distance. PC1 and PP1 represent feces from pregnant women in the control group and the probiotic group at the first sampling time, respectively; PC2 and PP2 represent feces from pregnant women in the control group and the probiotic group at the second sampling time CD1, CD3, CD14, and CM6 represent feces from infants in the control group at day 1, 3, 14, and month 6 after birth, respectively; PD1, PD3, PD14, and PM6 represent feces from infants in the probiotic group at day 1, 3, 14, and month 6 after birth, respectively.
